# Phospholipase Cγ2 Is Required for Luminal Expansion of the Epididymal Duct during Postnatal Development in Mice

**DOI:** 10.1371/journal.pone.0150521

**Published:** 2016-03-07

**Authors:** Hirotake Ichise, Taeko Ichise, Nobuaki Yoshida

**Affiliations:** Laboratory of Developmental Genetics, Center for Experimental Medicine and Systems Biology, The Institute of Medical Science, The University of Tokyo, Minato-ku, Tokyo, Japan; Clermont-Ferrand Univ., FRANCE

## Abstract

Phospholipase Cγ2 (PLCγ2)-deficient mice exhibit misconnections of blood and lymphatic vessels, and male infertility. However, the cell type responsible for vascular partitioning and the mechanism for male infertility remain unknown. Accordingly, we generated a mouse line that conditionally expresses endogenous *Plcg2* in a Cre/loxP recombination-dependent manner, and found that *Tie2-Cre-* or *Pf4-Cre*-driven reactivation of *Plcg2* rescues PLCγ2-deficient mice from the vascular phenotype. By contrast, male mice rescued from the vascular phenotype exhibited epididymal sperm granulomas. As judged from immunostaining, PLCγ2 was expressed in clear cells in the epididymis. PLCγ2 deficiency did not compromise differentiation of epididymal epithelial cells, including clear cells, and tube formation at postnatal week 2. However, luminal expansion of the epididymal duct was impaired during the prepubertal period, regardless of epithelial cell polarity and tube architecture. These results suggest that PLCγ2-deficient clear cells cause impaired luminal expansion, stenosis of the epididymal duct, attenuation of luminal flow, and subsequent sperm granulomas. Clear cell-mediated luminal expansion is also supported by the observation that PLCγ2-deficient males were rescued from infertility by epididymal epithelium-specific reactivation of *Plcg2*, although the edematous and hemorrhagic phenotype associated with PLCγ2 deficiency also caused spontaneous epididymal sperm granulomas in aging males. Collectively, our findings demonstrate that PLCγ2 in clear cells plays an essential role in luminal expansion of the epididymis during the prepubertal period in mice, and reveal an unexpected link between PLCγ2, clear cells, and epididymal development.

## Introduction

Phospholipase Cγ2 (PLCγ2) is a signaling molecule that is required for the normal differentiation and function of hematopoietic cells, including B lymphocytes, NK cells, mast cells, platelets, and osteoclasts [1–3]. Recently, we demonstrated that PLCγ2 also participates in tissue morphogenesis in mice [4]. We found that spontaneous and induced mutant mice lacking PLCγ2 exhibit lymphatic vessel abnormalities, such as chylous ascites and blood-filled lymphatic vessels, resulting from malfunctions in lymphatic circulation and misconnections between blood and lymphatic vessels. Our bone marrow transplantation experiments revealed that PLCγ2-deficient bone marrow-derived cells are involved in the pathogenesis of blood and lymphatic vessels. However, the cell type responsible for the vascular partitioning remains unknown.

In our previous study of the vascular phenotype, we found that PLCγ2-deficient males, but not females, are infertile, owing to azoospermia. Since adult PLCγ2-deficient mice are often severely affected by peritoneal bleeding due to the misconnection of blood and lymphatic vessels in visceral tissues, we speculated that the male reproductive system might be damaged by bleeding, or male germ cell development may be affected by inadequate heat exchange owing to a blood-lymph shunt in the male reproductive system. However, the pathogenesis of male infertility in PLCγ2-deficient mice is not clear, and it is not known whether male infertility is a secondary defect related to the vascular defect or not.

In this study, we generated a new mouse line that conditionally expresses endogenous *Plcg2* in a Cre/loxP recombination-dependent manner to identify the cell type responsible for the vascular phenotype. In the mouse line, endogenous *Plcg2* expression was silenced by the insertion of a *loxP*-flanked gene cassette in the 5′-untranslated region and reactivated by Cre-mediated excision of the cassette. We screened Cre-driver mouse strains that could rescue the mice from the vascular phenotype that results from a PLCγ2 deficiency and suggest that megakaryocytes are responsible for the vascular phenotype. Unexpectedly, we also found that male infertility was caused by sperm granuloma formation in the epididymis, and sperm granuloma formation was independent of the vascular defect. We focused on the role of PLCγ2 in the epididymis and identified PLCγ2-expressing clear cells of the epididymal duct as regulators of normal epididymal development.

## Results

### Generation of a genetically-engineered mouse line in which endogenous *Plcg2* is reactivated via Cre recombination

**Fig 1:**
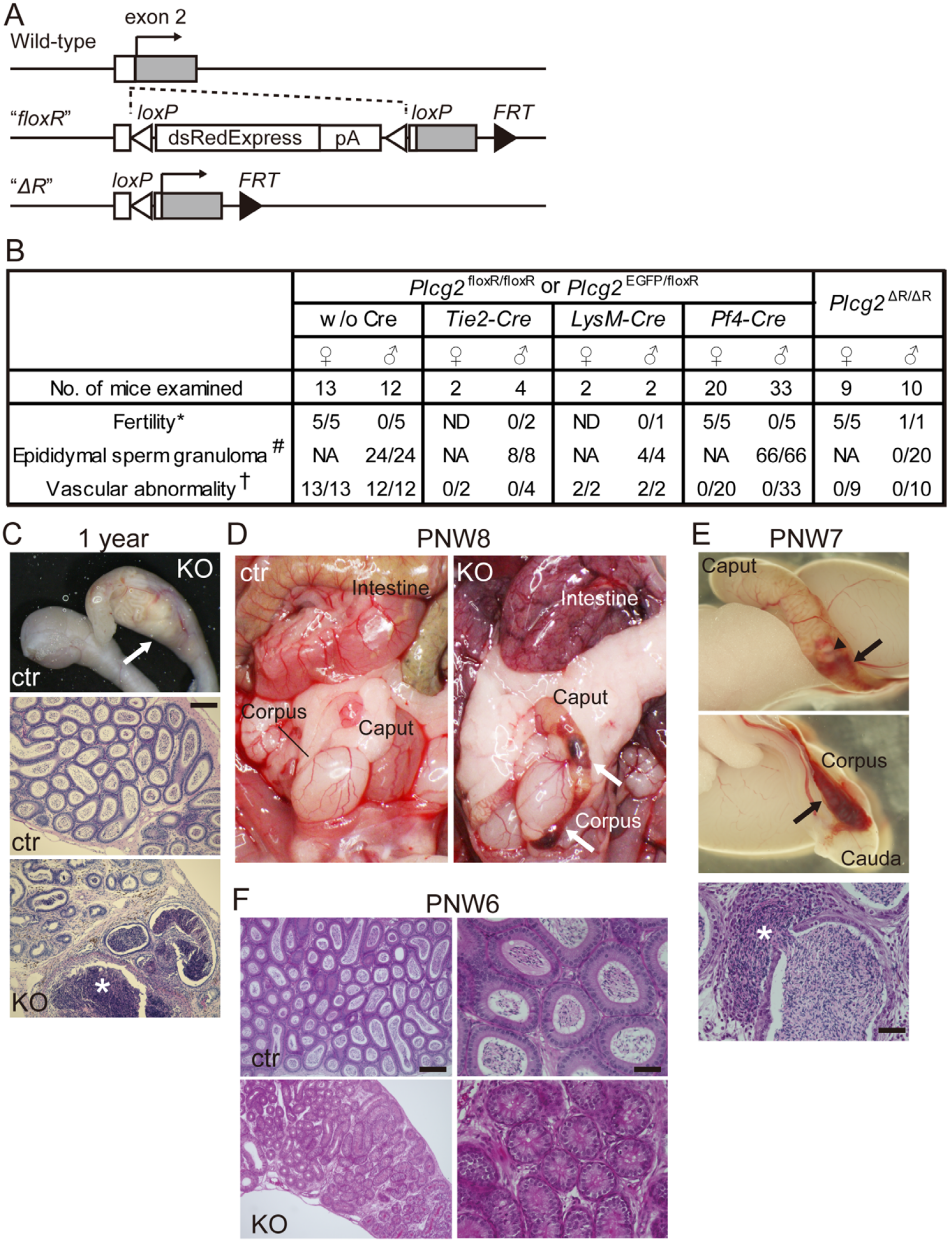
PLCγ2-deficient males exhibit epididymal sperm granulomas. (A) A schematic representation of *Plcg2*^floxR^, a genetically modified *Plcg2* allele that conditionally expresses *Plcg2* after Cre-mediated excision of a loxP-flanked dsRed-Express cassette in the 5′-untranslated region of exon 2. The FRT-flanked neo^r^ cassette was removed using a germline FLP-driver mouse line. (B) Summary of the phenotypic rescue analysis by using *Plcg2*^floxR^ knock-in mice with or without a Cre-driver allele. ND, not determined; NA, not applicable; * no. of mice that produced offspring of all mice mated for 3 months; # no. of epididymides harboring sperm granulomas from macroscopic findings; † no. of mice with blood-filled lymphatic vessels, tortuous blood vessels, or bleeding. (C) Epididymal sperm granuloma (arrow and asterisk) in the caput epididymidis of a 1-year-old PLCγ2-deficient male mouse. ctr, *Plcg2*^+/floxR^ male; KO, *Plcg2*^floxR/floxR^ male. Scale bar, 200 μm (for middle and bottom panels). (D) *Plcg2*^floxR/floxR^ males at postnatal week 8 (PNW8) exhibit tortuous blood vessel formation in the intestine and hemorrhaging in the distal caput and corpus epididymidis (arrows). ctr, *Plcg2*^+/floxR^ male; KO, *Plcg2*^floxR/floxR^ male. (E) *Plcg2*^floxR/floxR^ males at PNW7 have an epididymis bearing sperm granulomas (arrowhead in top panel) and bleeding (arrows in top and middle panels). Spermatozoa flow from ruptured epididymal ducts into the interstitial space (asterisk in bottom panel). Scale bar in bottom panel, 50 μm. (F) The distal caput region of the epididymis at PNW6. In PLCγ2-deficient mice, luminal narrowing is observed (lower panels). ctr, *Plcg2*^+/floxR^ male; KO, *Plcg2*^floxR/floxR^ male. Scale bars, 200 μm for left panels and 50 μm for right panels.

To determine the PLCγ2-expressing cell type responsible for the phenotypes observed in PLCγ2-deficient mice, we generated a genetically-engineered mouse line that harbors a modified *Plcg2* gene. In this line, we introduced *loxP*-sandwiched dsRed-Express cDNA followed by a polyadenylation (pA) signal sequence (abbreviated as floxR in the text below and figures) into the 5′-untranslated region upstream of the first translation initiation codon on the second exon of the *Plcg2* gene (Fig 1A). We did not detect any fluorescent signal of dsRed expression in the knock-in mice by fluorescence microscopy, in contrast to the *Plcg2*-EGFP knock-in mice that we have described previously [4]. This might be explained by the inefficient initiation of translation from the start codon juxtaposed with an upstream *loxP* sequence that consists of two inverted repeats in the allele. While the reporter cassette did not work properly, the homozygous knock-in mice showed the same phenotypes as PLCγ2-deficient mice [4] (Fig 1B), indicating that the dsRed-Express cDNA-pA cassette inactivated endogenous *Plcg2*. To examine whether the removal of the *loxP*-flanked cassette reactivates endogenous *Plcg2* expression, we removed the *loxP*-flanked cassette from the modified *Plcg2* allele by using the germline Cre-driver allele (the *Tie2-Cre* allele [5] in female germ cells) and obtained homozygous mice carrying the Cre-excised allele (*Plcg2*^ΔR^). The resulting *Plcg2*^ΔR/ΔR^ mice exhibited the wild-type phenotype (Fig 1B), thus demonstrating that the *loxP*-flanked cassette functions properly as a Cre-mediated binary switch of the *Plcg2* gene.

### *Pf4-Cre*-driven reactivation of endogenous *Plcg2* is sufficient for the normalization of vascular partitioning between blood and lymphatic vessels in PLCγ2-deficient mice

The majority of lymphatic vessels differentiate from blood vessels in embryos [6]. Lymphatic vessels are separated from blood vessels during embryonic development and are kept apart from blood vessels, except for a few direct connections between venous and lymphatic vessels, throughout postnatal life [7]. Genetic studies have suggested that correct vascular partitioning is megakaryocyte-dependent [8–11]. However, it has also been reported that endothelial progenitor cells and macrophages can participate in vascular partitioning [12, 13]. On the basis of those previous reports, we selected Cre-driver strains to turn on the *Plcg2* gene in particular cell types: *Tie2-Cre* in endothelial and hematopoietic cells [5], *LysM-Cre* in macrophages [14], and *Pf4-Cre* in megakaryocytes and a subset of hematopoietic stem cells [15, 16]. We checked blood-filled lymphatic vessels and tortuous blood vessels in the intestines of adult *Plcg2*^floxR/floxR^ or *Plcg2*^EGFP/floxR^ mice carrying either Cre-driver allele. Our results clearly showed that *Plcg2* reactivation by *Tie2-Cre* and *Pf4-Cre* was sufficient for the normalization of vascular partitioning in PLCγ2-deficient mice, as summarized in Fig 1B. Previous studies have suggested that the partitioning process begins with the interaction between C-type lectin receptor-2 (CLEC-2)-positive platelets and lymphatic endothelial cells expressing a binding partner of CLEC-2, *O*-glycosylated podoplanin [17], followed by CLEC-2-induced Syk and SLP-76 activation in platelets [8, 10, 11]. Our results suggest that PLCγ2 is an essential effector of podoplanin-induced CLEC-2 signaling in megakaryocytes and platelets during the vascular partitioning process, although it remains unclear whether *Pf4-Cre*-induced *Plcg2* activation outside the megakaryocyte lineage contributes to the rescue of the vascular phenotype or not [16].

### Epididymal sperm granulomas develop independently of blood-lymphatic vessel misconnections and bleeding in PLCγ2-deficient male mice

As described briefly in the Introduction, PLCγ2-deficient males are infertile. In detail, PLCγ2-deficient male mice never produce offspring, irrespective of copulatory plug formation. At necropsy, nodular lesions and bleeding are seen in the epididymis of adult PLCγ2-deficient male mice, and no spermatozoa can be collected from cauda epididymides. These observations suggest that the observed male infertility is related to epididymal anomalies; however, it is not clear how epididymides are affected by the absence of PLCγ2.

**Fig 2:**
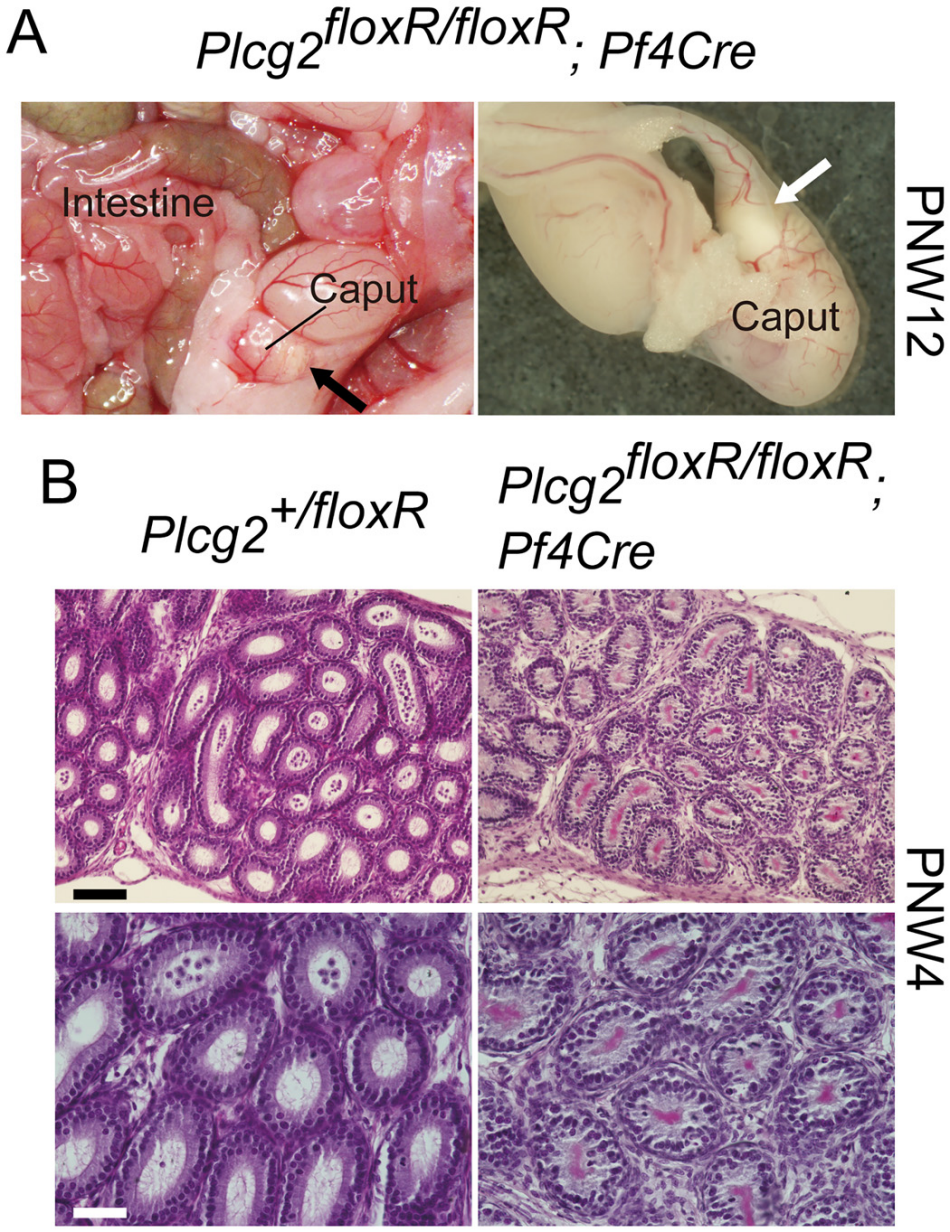
PLCγ2 expression in megakaryocytes rescues PLCγ2-deficient males from vascular phenotypes but not epididymal sperm granulomas. (A) *Plcg2*^floxR/floxR^;*Pf4-Cre* males are rescued from tortuous blood vessel formation in the intestine but still exhibit epididymal sperm granulomas (arrows). (B) Luminal narrowing is observed in the epididymis of *Plcg2*^floxR/floxR^;*Pf4-Cre* males at PNW4. Scale bars, 100 μm for upper panels and 50 μm for lower panels.

An epididymis has a long, highly convoluted epithelial tube, known as the epididymal duct. The proximal and distal ends of the duct are connected to the efferent ducts and vas deferens, respectively. Spermatozoa and testicular fluid flow from the testis into the epididymis via efferent ducts, and flow out through the vas deferens. In adults, spermatozoa leaving the testis acquire motility and fertilization ability when passing through the epididymal duct, and become fully matured and concentrated in the distal epididymis (reviewed in [18]). We examined the epididymis of adult PLCγ2-deficient male mice histologically and found that the nodular lesions in the proximal epididymis represent epididymal sperm granulomas, which are dumps of extravasated spermatozoa surrounded by granulomatous tissue in the interstitial space of the epididymis (Fig 1C). Epididymal sperm granulomas in hemorrhaged epididymides, as well as the vascular abnormality in the intestine, were obvious in young adult males lacking PLCγ2 (Fig 1D and 1E). We found epididymal sperm granulomas in all PLCγ2-deficient male mice at postnatal week (PNW) 7 and later (Fig 1B and 1E). Before granulomatous tissue formation around spermatozoa, we observed leakage of spermatozoa from ruptured epididymal ducts in the distal caput epididymidis (Fig 1E). Six-week-old PLCγ2-deficient males did not exhibit epididymal sperm granulomas at the gross morphological level, but exhibited abnormal ducts with narrow lumen in the distal caput epididymidis (Fig 1F). We speculated that the epididymal ducts in PLCγ2-deficient males were damaged by edematous conditions owing to a blood-lymph shunt because edema in the epididymis can cause sperm granulomas [19]. However, sperm granuloma formation was not blocked by the normalization of the vascular phenotype in *Plcg2*^floxR/floxR^ mice (or *Plcg2*^EGFP/floxR^ mice) carrying *Tie2-Cre* or *Pf4-Cre* (Figs 1B and 2A). Furthermore, 4-week-old *Plcg2*^floxR/floxR^;*Pf4-Cre* males did not exhibit epididymal sperm granulomas but already exhibited abnormal ducts with narrow lumen in the distal caput epididymidis (Fig 2B). Although these results do not exclude the possibility that vascular phenotypes prior to *Plcg2* activation by Cre in embryos and postnatal mice lead to epididymal phenotypes, these results suggested the involvement of cells other than endothelial and blood cells in determining epididymal phenotypes. Therefore, we sought PLCγ2-expressing cells in the epididymis, and examined their role in epididymal morphogenesis and function.

### PLCγ2 is expressed specifically in clear cells in the epididymis

The epididymal duct consists of three major types of epithelial cells. Principal cells are the main epithelial cells in the epididymal duct, and absorb and secrete luminal proteins (reviewed in [18]). Basal cells act as luminal pH sensors to regulate clear cell secretion of protons into the lumen [20]. Clear cells, and narrow cells in the proximal caput, lead to the acidification of the luminal fluid in response to signals from basal cells and principal cells [20], and also take up components of disrupted cytoplasmic droplets released into the epididymal lumen [21]. Together with these epithelial cells, myoid cells and immune cells contribute to epididymal duct formation and function (reviewed in [18]).

**Fig 3:**
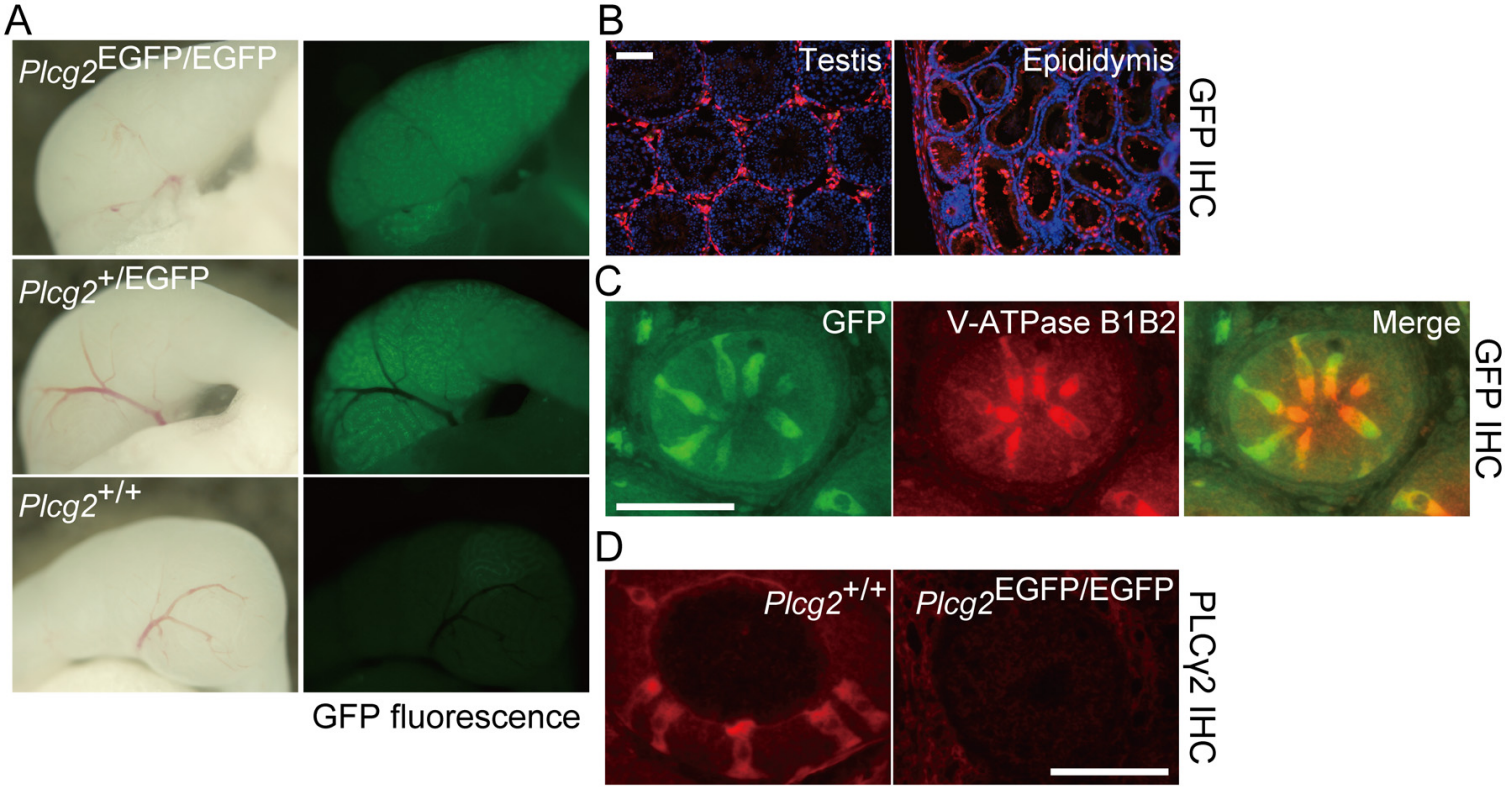
PLCγ2 is expressed in clear cells of the epididymis. (A) *Plcg2*-driven EGFP is expressed in the epididymis of mice at PNW4. The caput epididymidis is shown. (B) GFP-immunostained sections of an adult *Plcg2*^EGFP/+^ male. In the testis, GFP is expressed in interstitial cells, but not in male germ lineages in seminiferous tubules (left). In the epididymis, a subset of epididymal epithelial cells are GFP-positive (right). Epididymal ducts in the caput epididymidis are shown. Scale bar, 100 μm. (C) Double immunostaining for GFP and V-ATPase B1/B2. *Plcg2*-driven EGFP expression is found in V-ATPase B1/B2-positive clear cells. The epididymal duct in the distal caput epididymidis of a *Plcg2*^EGFP/EGFP^ male at PNW4 is shown. Scale bar, 50 μm. (D) PLCγ2 immunostaining also detects clear cells in the epididymal epithelial cell layer. Scale bar, 50 μm.

To determine the *Plcg2* expression pattern in the epididymis, we examined EGFP expression in *Plcg2*-EGFP knock-in mice. The epididymal ducts of those mice were GFP-positive in fluorescence microscope (Fig 3A), and by using immunohistochemistry, we found that *Plcg2*-driven EGFP is expressed in V-ATPase-positive clear cells in the epididymal duct (Fig 3B and 3C). We obtained a consistent result by using PLCγ2 immunostaining (Fig 3D). Spermatozoa and their progenitors in seminiferous tubules in the testes did not express PLCγ2 (Fig 3B). In PLCγ2-deficient mice, clear cells developed normally, as judged by the immunostaining results for EGFP and V-ATPase subunits (Fig 3C).

### The lumen of the epididymal duct does not expand in PLCγ2-deficient male mice during the prepubertal period

The epididymal duct in the distal caput and corpus epididymidis has a pseudostratified epithelial cell layer that includes Aquaporin 9 (Aqp9)-expressing principal cells [22], V-ATPase B1/B2 subunit-positive clear cells [23], and keratin 5 (K5)-positive basal cells [24], and an outer layer of alpha smooth muscle actin (αSMA)-positive myoid cells. Like other types of epithelial tubes, the integrity of the epididymal duct is maintained by epithelial polarity, tight junctions (TJs), and adherens junctions (AJs) (reviewed in [18]). Polarized epithelial cells have an apical surface characterized by the localization of atypical protein kinase C (aPKC). The formation of TJs and AJs can be characterized by the localization of zonula occludens-1 (ZO-1) and β-catenin, respectively. Aqp9 also localizes at the apical surface of principal cells. TJ and AJ formation between epididymal epithelial cells is thought to be essential for the maintenance of the luminal microenvironment and formation of the blood—epididymis barrier. We evaluated epithelial cell differentiation, tube structure, and the integrity of the duct by immunostaining for the cell markers, aPKC, ZO-1, and β-catenin.

As described above, hematoxylin and eosin (HE)-stained sections showed luminal narrowing of epididymal ducts in the caput epididymides of *Plcg2*^floxR/floxR^;*Pf4-Cre* males at PNW4 (Fig 2B). Attenuation of luminal flow owing to a smaller lumen can also lead to attenuation of luminal factor-dependent epididymal development. Therefore, we examined younger male mice to elucidate the primary defect associated with PLCγ2 deficiency.

**Fig 4:**
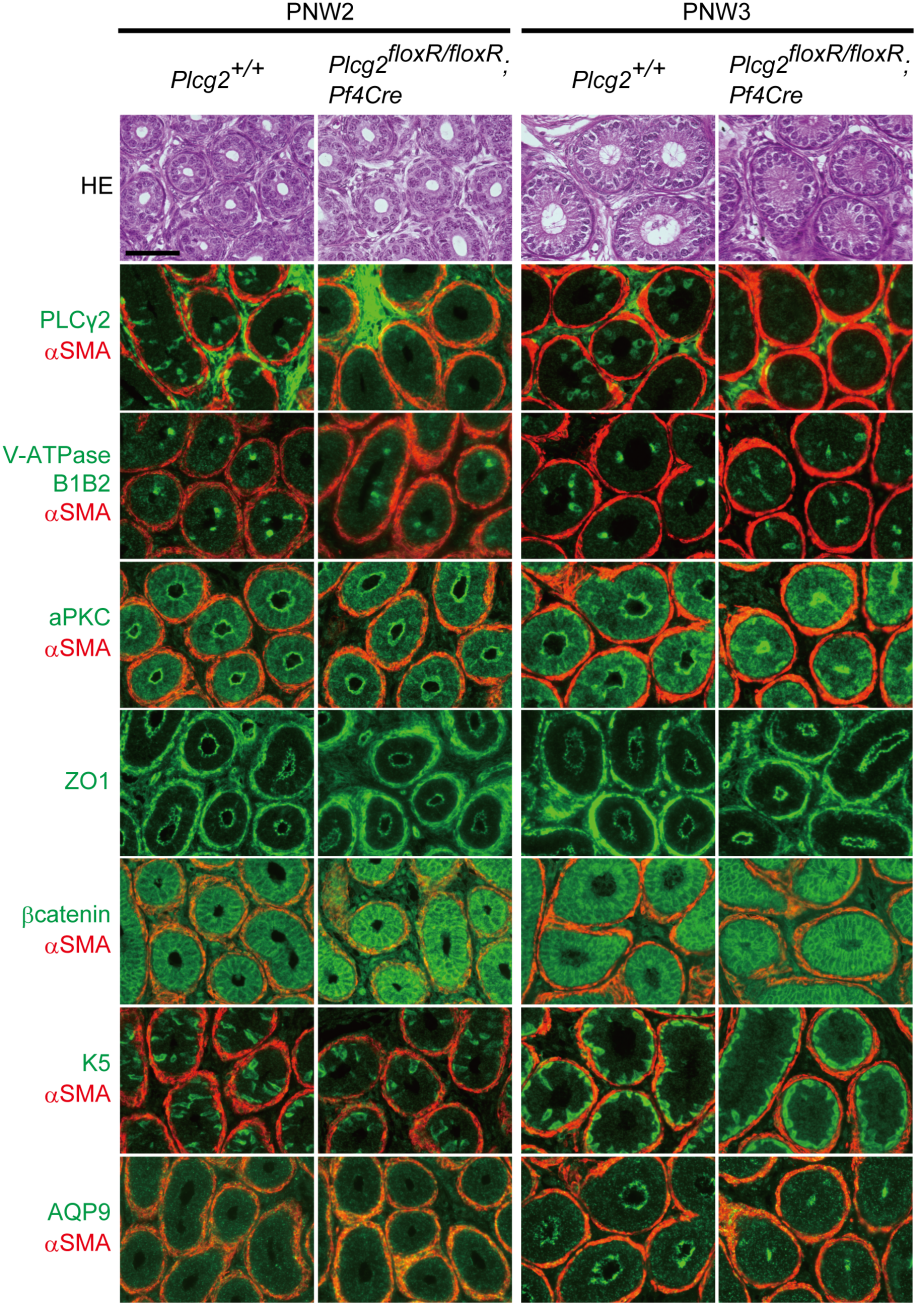
Epididymal ductal development in the distal caput epididymidis of wild-type and *Plcg2*^floxR/floxR^;*Pf4-Cre* males at PNW2 and PNW3. HE, hematoxylin and eosin staining; V-ATPase B1B2, clear cell marker; aPKC, apical surface marker; ZO1, tight junction marker; β-catenin, adherens junction marker; K5, basal cell marker; Aqp9, marker for apical surface of principal cells; αSMA, myoid cell marker. Aqp9 on the apical surface is not detected in the distal caput at PNW2. Signals in the interstitial tissue of **PLC**γ**2**-immunostained sections are background staining. Scale bar, 50 μm.

The apical surface, TJs and AJs formed at PNW2 and PNW3, as demonstrated by immunostaining for aPKC, and ZO-1, and β-catenin (Fig 4), indicated that epithelial polarity, TJs and AJs were not affected in PLCγ2-deficient mice. By contrast, immunostaining for Aqp9, aPKC, and ZO-1 showed that the apical surface of the duct in the distal caput and corpus at PNW3 exhibited extreme shrinkage, regardless of apical surface formation (Fig 4). However, the distributions of clear cells, basal cells, and myoid cells in *Plcg2*^floxR/floxR^;*Pf4-Cre* males were comparable to those in control males (Fig 4). These results suggest that epithelial cell type differentiation and duct formation are not affected by PLCγ2 deficiencies in males.

**Fig 5:**
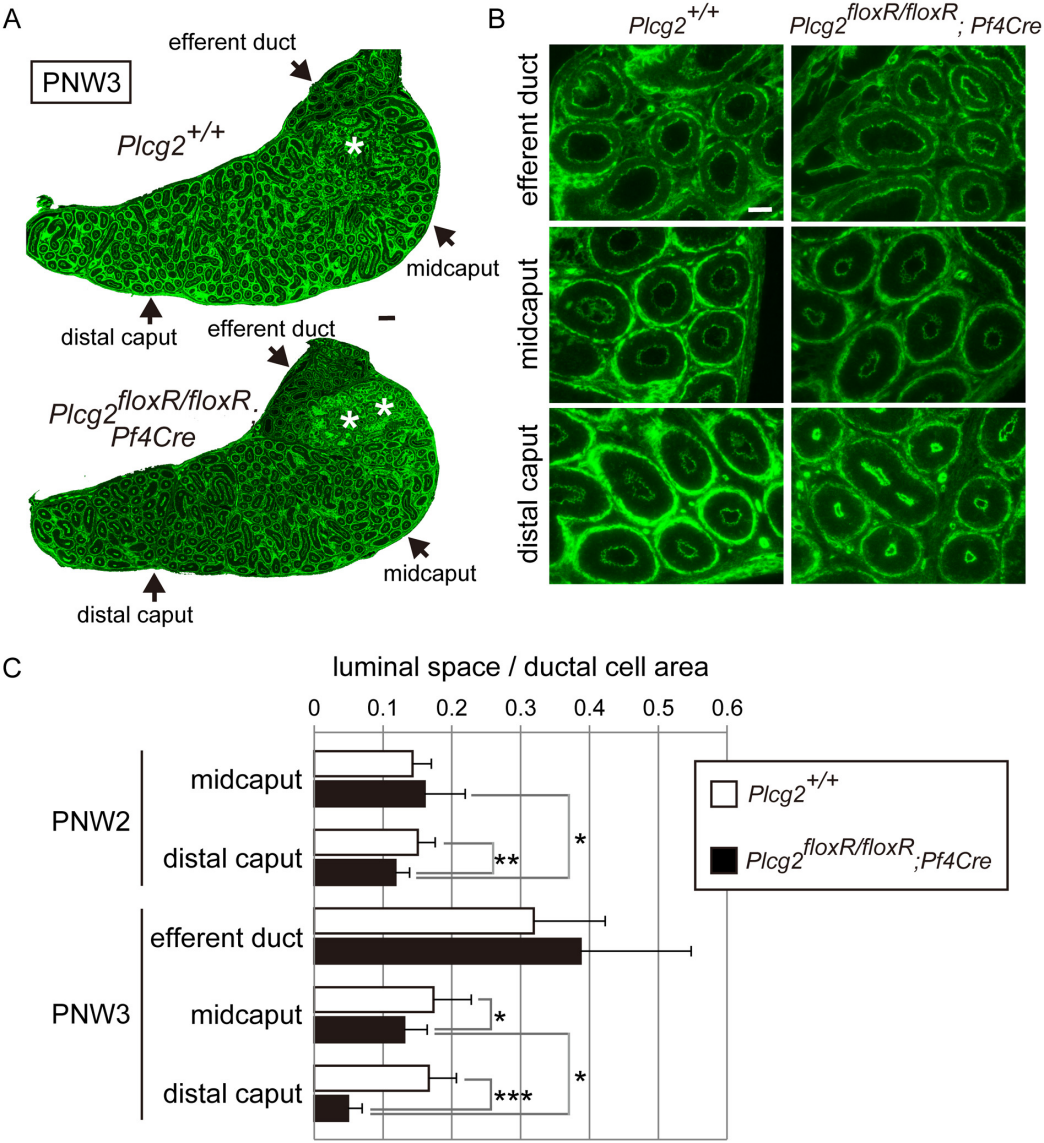
Luminal narrowing of the duct in the distal caput epididymidis in *Plcg2*^floxR/floxR^;*Pf4-Cre* males. (A) Merged images of multiple micrographs showing whole ZO-1-immunostained sections of the caput epididymidis with efferent ducts at PNW3. Asterisks, high background signals in subsets of ductal cells and luminal compounds in the initial segment. Scale bars, 100 μm. (B) Efferent ducts and epididymal ducts in the midcaput and distal caput epididymidis in the ZO-1-immunostained sections. Scale bar, 50 μm. (C) Quantification of the ratio of luminal space to ductal cell area of ducts in different regions at PNW2 and PNW3. n = 30 (ten ducts per epididymis of three epididymides). * p < 1 × 10^-3^, ** p < 1 × 10^-5^, *** p < 1 × 10^-21^.

To quantitatively assess luminal narrowing, we measured the luminal space of ducts in three different domains, i.e., efferent ducts and epididymal ducts in the midcaput and distal caput, on the ZO-1-immunostained sections (Fig 5A and 5B). At PNW2, we detected a slight narrowing of the duct in the distal caput of *Plcg2*^floxR/floxR^;*Pf4-Cre* males, but we did not observe a significant difference in the ratio of luminal space to epithelial cell area of ducts in the midcaput between *Plcg2*^floxR/floxR^;*Pf4-Cre* and control males (Fig 5C). At PNW3, the ratio in the epididymal duct was significantly smaller in *Plcg2*^floxR/floxR^;*Pf4-Cre* males than control males, but the ratio in the efferent ducts of *Plcg2*^floxR/floxR^;*Pf4-Cre* males was comparable to that of control males (Fig 5C). These results suggest that the maintenance of the luminal space in the distal part of the caput epididymidis is affected by a PLCγ2 deficiency in males during epididymal development between PNW2 and PNW3. PLCγ2-deficient clear cells may play a role in preventing luminal narrowing during epididymal development.

### PLCγ2 in clear cells is required for luminal expansion of the epididymal duct during postnatal development

**Fig 6:**
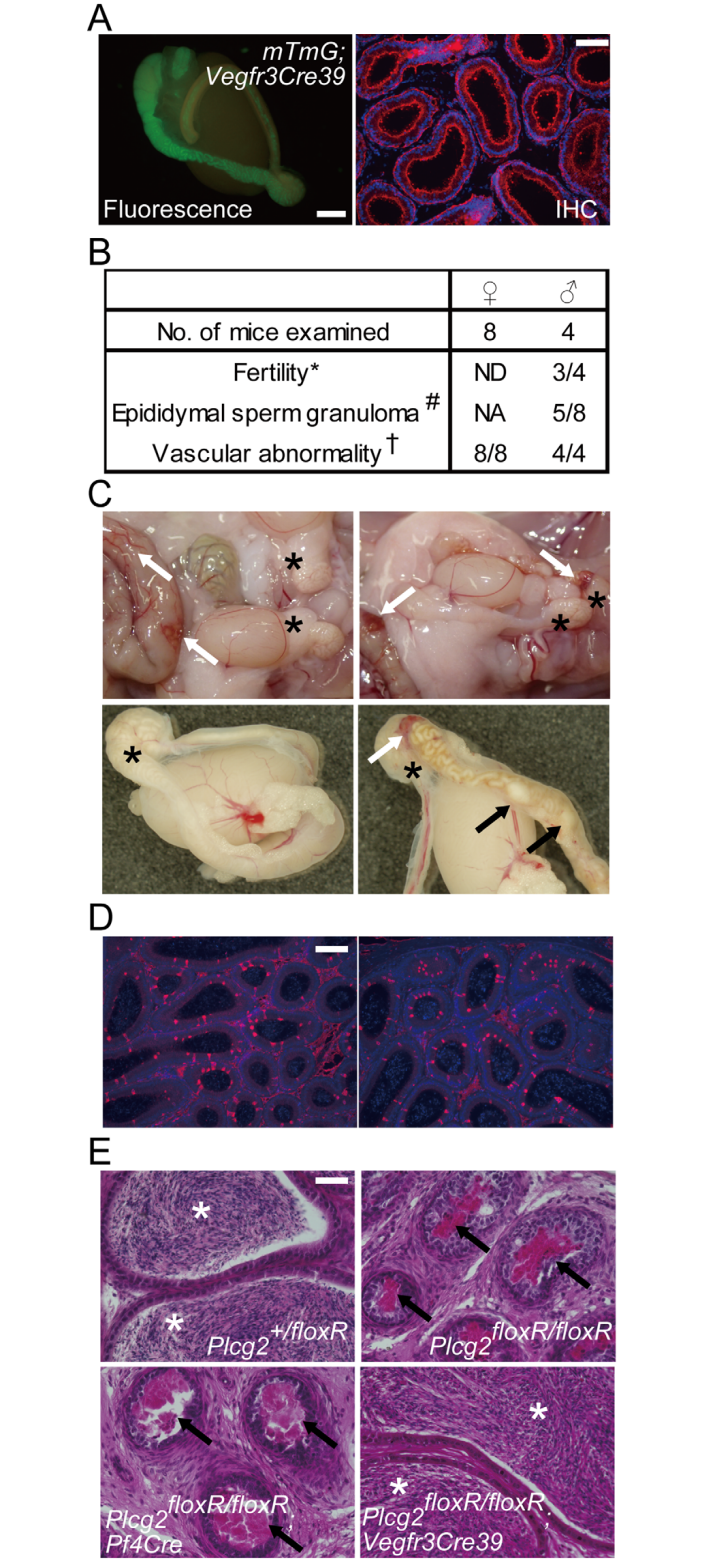
Normal development and function of the epididymis are required for PLCγ2 expression in clear cells. (A) *Vegfr3-Cre*39 drives Cre recombination in epididymal epithelial cells. GFP fluorescence in the epididymis of an mT/mG;*Vegfr3-Cre*39 male at PNW3 (left panel) and GFP immunostaining of the duct in the caput epididymidis of an mT/mG;*Vegfr3-Cre*39 male at PNW12 (red in right panel) are shown. Scale bars for left and right panels, 1 mm and 100 μm, respectively. (B) Summary of the phenotypic rescue analysis in *Plcg2*^floxR/floxR^;*Vegfr3-Cre*39 and *Plcg2*^EGFP/floxR^;*Vegfr3-Cre*39 mice. ND, not determined; NA, not applicable; * no. of mice that produced offspring of all mice mated for 3 months; # no. of epididymides harboring sperm granulomas in macroscopic findings; † no. of mice with blood-filled lymphatic vessels, tortuous blood vessels, or bleeding. (C) Six-month-old *Plcg2*^floxR/floxR^;*Vegfr3-Cre*39 males exhibit the vascular phenotype, such as tortuous blood vessels in the intestine, blood-filled lymphatic vessels in the Payer’s patch, and hemorrhaging in the epididymis (white arrows), and spontaneous epididymal sperm granulomas (black arrows). However, they also have normal epididymides (asterisks on the cauda epididymidis). Right and left panels represent epididymides with and without sperm granulomas, respectively. (D) PLCγ2 immunostaining of the duct in the distal caput epididymidis of 6-month-old *Plcg2*^floxR/floxR^;*Vegfr3-Cre*39 males with and without sperm granulomas in the corpus epididymidis (right and left panels, respectively). PLCγ2 expression in clear cells and luminal expansion are recovered in *Plcg2*^floxR/floxR^;*Vegfr3-Cre*39 males. Signals in the interstitial tissue are background staining. Scale bar, 100 μm. (E) *Plcg2*^floxR/floxR^;*Vegfr3-Cre*39 males as well as control males (*Plcg2*^+/floxR^) store spermatozoa in the caudal region of the epididymis (asterisks), while *Plcg2*^floxR/floxR^ males and *Plcg2*^floxR/floxR^;*Pf4-Cre* males have poorly expanded ducts and their spermatozoa do not reach the caudal epididymidis (arrows).

To verify that PLCγ2 directs luminal expansion in a clear cell-dependent manner, we performed a rescue experiment in a Cre-driver mouse line for epididymal epithelial cells. *Vegfr3-Cre*39, as well as *Vegfr3-Cre*44 described in a previous study [25], was generated by using a *Vegfr3* promoter-driven transgene via random integration in fertilized eggs to obtain an endothelial cell-specific Cre-driver. Line 39 was not a good endothelial cell-specific Cre-driver, but showed ectopic Cre expression in non-endothelial cells, such as cardiomyocytes and epididymal epithelial cells (Fig 6A), probably due to a position effect of the integrated transgene. Using this line, we examined whether epididymal epithelial cell-specific reactivation of PLCγ2 could rescue PLCγ2-deficient mice from impaired lumen expansion and azoospermia. Three out of four *Plcg2*^floxR/floxR^;*Vegfr3-Cre*39 males were fertile when they were young (Fig 6B) but became poor breeders over time. At 6 months after birth, we often detected sperm granulomas in the corpus epididymidis of 6–11-month-old *Plcg2*^floxR/floxR^;*Vegfr3-Cre*39 males (Fig 6B and 6C). One out of four *Plcg2*^floxR/floxR^;*Vegfr3-Cre*39 males did not produce offspring (Fig 6B) and exhibited sperm granulomas in the corpus epididymidis. *Vegfr3-Cre*39-driven *Plcg2* reactivation in those mice with or without sperm granuloma formation was verified by immunostaining for PLCγ2 (Fig 6D). Edematous conditions in the epididymis can lead to sperm granuloma formation [19], and blood and lymphatic vessel malfunction and bleeding in the epididymides of these mice may also cause spontaneous sperm granuloma formation. Nevertheless, the cauda epididymidis of 6–11-month-old *Plcg2*^floxR/floxR^;*Vegfr3-Cre*39 males, with or without sperm granulomas, were well developed and the lumen contained spermatozoa with elongated nuclei, in sharp contrast to those of 8-week-old *Plcg2*^floxR/floxR^ and *Plcg2*^floxR/floxR^;*Pf4-Cre* males (Fig 6E).

**Fig 7:**
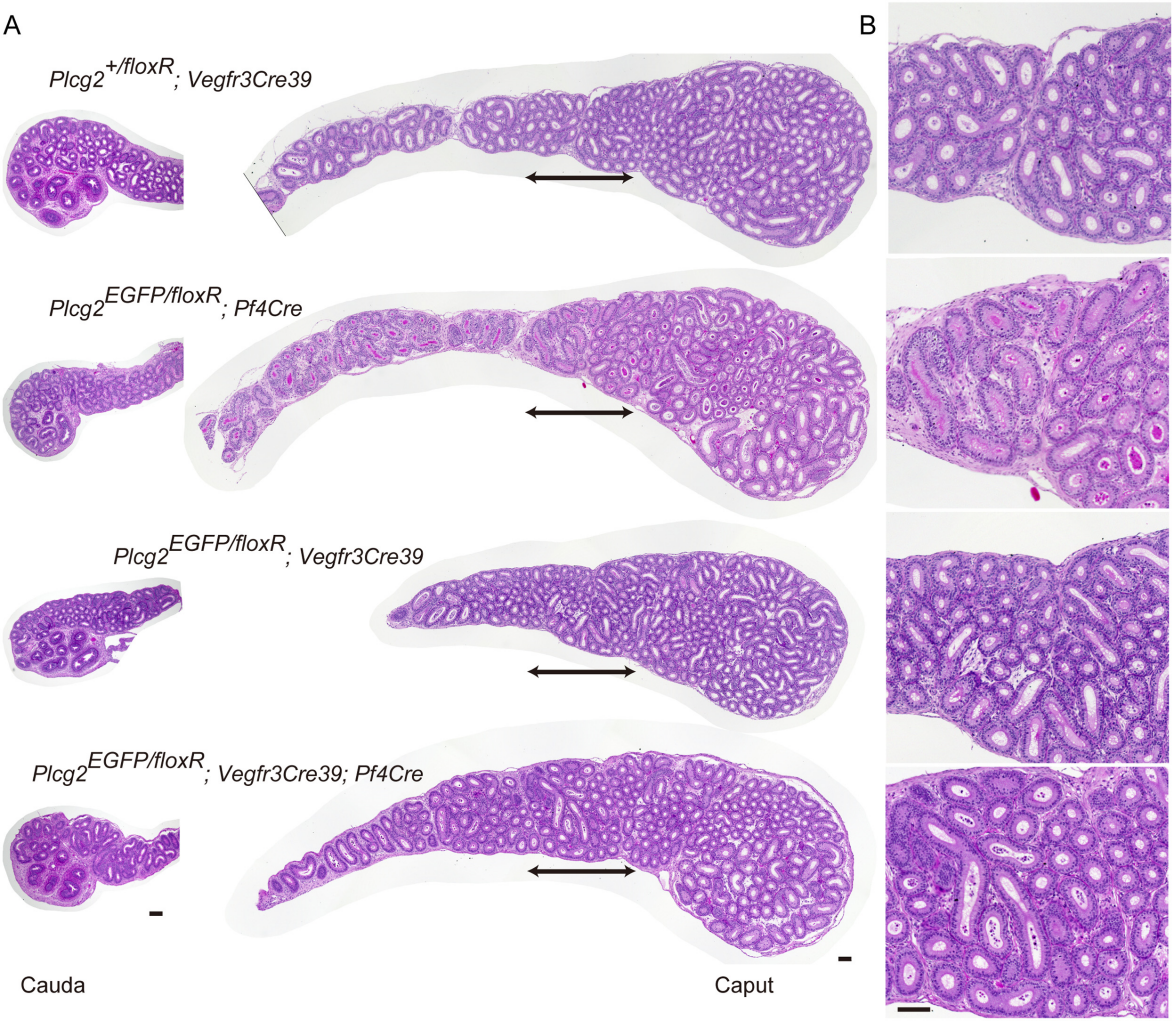
Luminal space recovery of the epididymal duct by PLCγ2 expression in clear cells of PLCγ2-deficient males. (A) Merged images of multiple micrographs showing whole HE-stained sections of the caput epididymidis (middle) and micrographs showing the cauda epididymidis (left) at PNW4 in males. Scale bars, 100 μm. (B) Magnified images of the distal caput epididymidis indicated in (A). Scale bar, 100 μm.

However, it is very unlikely that ruptured epididymal ducts regenerate and regain their function in fertility. The elongated nuclei of spermatozoa found in the cauda of affected males may reflect a history of sperm transport through epididymal ducts at an earlier stage. Some males produced offspring when young, but did not exhibit sustained fertility over time; accordingly, it is likely that they develop sperm granulomas after reaching puberty, and sperm granuloma formation occurs later than it does in *Plcg2* KO males. To elucidate this possibility, we examined the epididymis of *Plcg2*^EGFP/floxR^;*Vegfr3-Cre*39 males at PNW4 (Fig 7). We found that, in contrast to *Plcg2*^EGFP/floxR^;*Pf4-Cre* males, a *Plcg2*^EGFP/floxR^;*Vegfr3-Cre*39 male and a *Plcg2*^EGFP/floxR^;*Vegfr3-Cre*39;*Pf4-Cre* male at PNW4 had epididymides with expanded lumen (Fig 7). This observation suggests that the epididymides of young *Plcg2*^EGFP/floxR^;*Vegfr3-Cre*39 males are able to transport luminal fluid and spermatozoa before sperm granuloma formation later in life.

**Fig 8:**
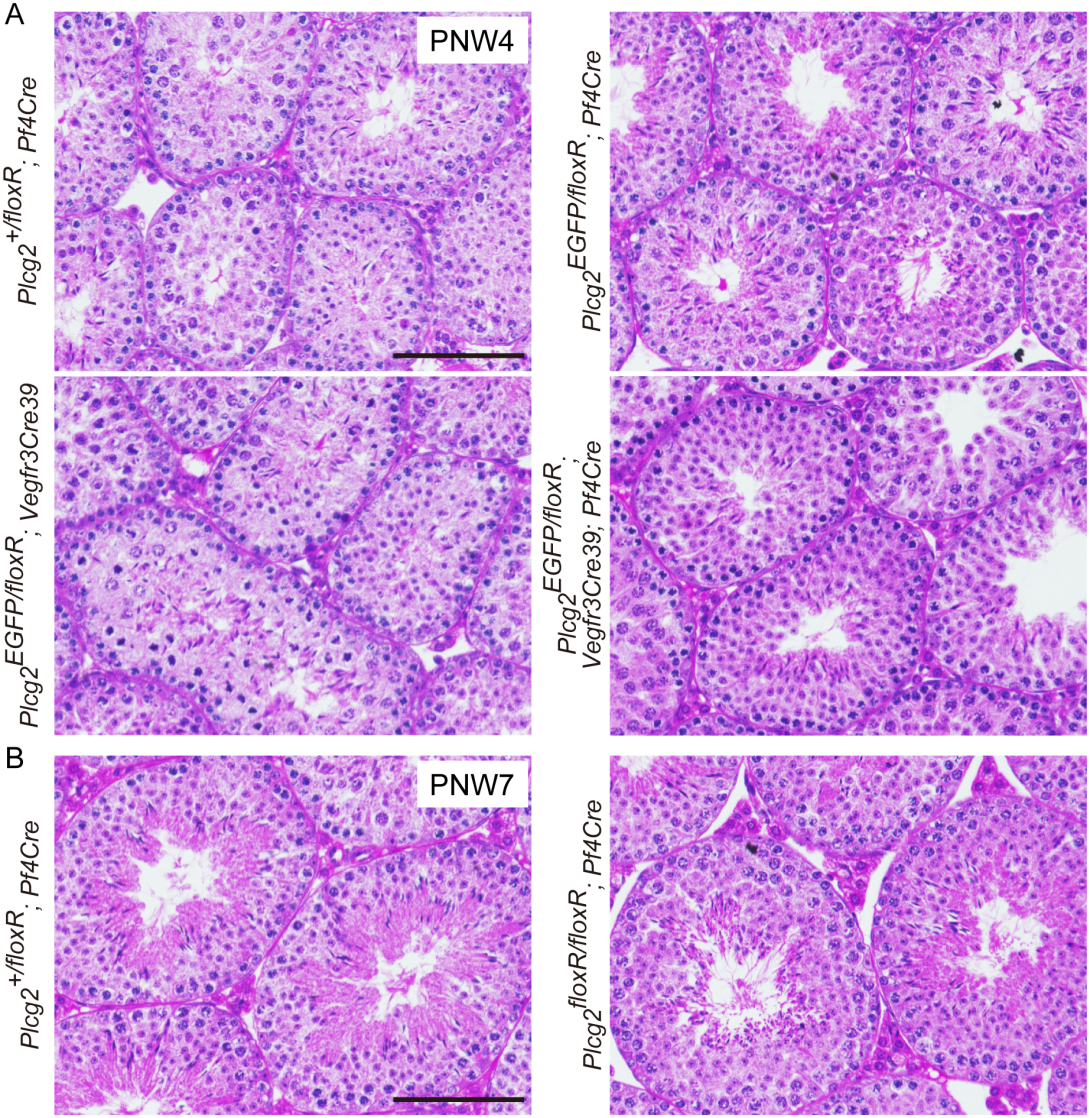
Normal spermatogenesis in the testis of PLCγ2-deficient males. Sections of the testis at PNW4 (A) and PNW7 (B) are shown. Elongated nuclei of spermatids are seen in the seminiferous tubules. Scale bars, 100 μm.

Testicular fluid reduction and defective spermatogenesis might also cause luminal narrowing. In control and mutant males, we found normal spermatogenesis in all the testes examined at PNW4 and PNW7 (Fig 8). The normal spermatogenesis and normal luminal space of efferent ducts, as shown in Fig 5, suggest that mutant young males do not have testicular fluid reduction.

Even though sperm granulomas develop spontaneously in pubertal *Plcg2*^floxR/floxR^;*Vegfr3-Cre*39 males and result in male subfertility, it is probable that PLCγ2-deficient clear cells are primarily responsible for luminal narrowing of the epididymal duct during the prepubertal period.

## Discussion

We have provided evidence for a PLCγ2-dependent role of clear cells in luminal expansion of the epididymal duct. We found that PLCγ2 expression in the epididymal duct is restricted to clear cells and PLCγ2 deficiency in these cells leads to male infertility. We suggest a model for the pathogenesis of male infertility in PLCγ2-deficient males as follows: the luminal space of the epididymal duct from the distal caput to the distal cauda epididymidis is severely reduced in size by malfunctions in PLCγ2-deficient clear cells, attenuating luminal flow and luminal factor-dependent maturation of the epididymal duct; movement of spermatozoa to the cauda epididymidis is also attenuated; as a result, breakdown of the epididymal duct and formation of sperm granulomas occur in the distal cauda and corpus epididymidis, resulting in infertility caused by azoospermia.

Clear cells take up components released by sperm cytoplasmic droplets during sperm maturation in the epididymal lumen [21]. These cells also lead to the acidification of the luminal fluid in response to signals from basal cells, and thereby maintain a suitable luminal environment for sperm maturation and storage [20, 26]. Another study has also suggested an essential role of clear cells in the regulation of the luminal environment in the epididymis. The transcription factor Foxi1 is expressed in narrow and clear cells, is necessary for the expression of ion transporters (such as the B1-subunit of the V-ATPase, carbonic anhydrase II, and pendrin), and is required for sperm maturation in the epididymis and male fertility [27]. Clear cells acquire the expression of ion transporters in a Foxi1-dependent manner at 15 days after birth, suggesting that they begin functioning around PNW2. Luminal abnormality of PLCγ2-deficient males at PNW3, but not PNW2, may be related to clear cell differentiation and maturation.

In contrast to the roles of clear cells in sperm maturation reported in previous studies, our findings suggest a distinct role of clear cells in morphogenesis of the epididymal duct. It is not known how PLCγ2-deficient clear cells lead to luminal narrowing. Generally, luminal expansion of epithelial tubes requires an asymmetric ion distribution between cells and the lumen, and fluid accumulation into the lumen. One explanation for the pathogenesis is that the clear cell-dependent ion transport system may be defective in PLCγ2-deficient males. A recent study has demonstrated that a solute carrier 26 (Slc26) family protein is indispensable for luminal expansion, but not for apical-luminal membrane formation in *Ciona intestinalis* [28]. One Slc26 family protein, Slc26a4 (also known as pendrin), is expressed in clear cells in a Foxi1-dependent manner [27]. Although Foxi1-deficient males that lack expression of Slc26a4 in clear cells do not exhibit luminal narrowing, dysregulation of Slc26 family proteins in the absence of PLCγ2 is a potential cause of luminal narrowing.

However, the ratio of the luminal space to the ductal cell area remained unchanged between epididymal ducts at PNW2 and PNW3 in controls (Fig 5), suggesting that luminal expansion is not induced by clear cells during this stage. In addition, lumen formation and initial luminal expansion in epididymal tube morphogenesis appeared normal because we observed the lumens of ducts in PLCγ2-deficient males at PNW2 (Fig 5). Thus, the results suggest that PLCγ2-deficient clear cells impair the maintenance of luminal size, rather than increase the luminal size, at PNW3. Luminal expansion of the epididymal duct depends on both luminal opening and increased luminal flow. We postulate that clear cells participate in luminal expansion by keeping the lumen opened, promoting luminal flow.

Zhao et al. reported that Bmp8a-deficient mutants exhibit epithelial degeneration and subsequent sperm granulomas in the distal caput and cauda epididymidis, while Bmp8a is expressed in the initial segment of the caput epididymidis [29]. The report suggests that either Bmp8a or another humoral factor produced in response to Bmp8a acts as a paracrine factor in epithelial cells downstream of the initial segment to maintain the integrity of the duct. Similarly, sperm granulomas develop in the distal segments of the epididymis of SED1/MFG-E8-deficient mice, even though SED1/MFG-E8 is secreted by the initial segment of the epididymis [30]. Given that luminal flow supplies humoral factors essential for the maintenance of epithelial integrity, it is likely that defective luminal expansion results in the depletion of the proximal epididymis-derived humoral factors in luminal fluid, and the subsequent degeneration and breakdown of epithelial cells in PLCγ2-deficient mice.

Ethyl nitrosourea-induced mutant mouse alleles, *Ali5* and *Ali14*, are gain-of-function *Plcg2* alleles, whose mutated PLCγ2 proteins lead to hyper-reactive external entry of calcium into cells in vitro [31, 32]. These alleles, in an autosomal dominant manner, cause inflammatory arthritis, metabolic defects, and male reproductive phenotypes, such as complete male infertility of *Plcg2*^*Ali5/+*^ mice in vivo [32] and extremely low in vitro fertilization rates with spermatozoa from *Plcg2*^*Ali14/+*^ mice that show normal fertility in vivo [32]. Haploid spermatozoa carrying wild-type *Plcg2*, as well as those carrying *Plcg2*^*Ali14*^ from *Plcg2*^*Ali14/+*^ mice, show impaired fertility in vitro; accordingly, Abe et al. speculated that mutated PLCγ2 is expressed in diploid spermatogonial stem cells, distributed to mature spermatozoa, and involved in the acrosome reaction of spermatozoa. However, the PLCγ2 expression pattern in the male reproductive system was not examined in that study. Because we observed PLCγ2 expression in clear cells of the epididymis, but not spermatozoa or their progenitors, as described above (Fig 3B), it is possible that hyper-reactive PLCγ2 proteins alter the function of clear cells in the epididymis and compromise the maturation of spermatozoa in the epididymal lumen.

It is necessary to determine how PLCγ2-mediated signaling functions in clear cells during lumen expansion in future studies; however, our findings on the close link between PLCγ2, clear cells, and epididymal development provide a basis for studies of the morphogenesis and function of the epididymis.

## Materials and Methods

### Mice and ethics statement

C57BL/6J and MCH:ICR mice were purchased from CLEA Japan (Tokyo, Japan). *Tie2-Cre* [5], *LysM-Cre* [14], *Pf4-Cre* [15], and mT/mG mice [33] were obtained from the Jackson Laboratory (Bar Harbor, ME, USA). FLP66 transgenic mice (RBRC01252) [34] were obtained from RIKEN BRC (Tsukuba, Japan). The generation of *Vegfr3-Cre* transgenic lines, including the *Vegfr3-Cre*39 line used in this study, was described previously [25]. All mice were housed under pathogen-free conditions. All mouse work in this study was approved by the Animal Care and Use Committee of the University of Tokyo and was conducted in accordance with their guidelines (approval nos. 19–13, 19–14, PA11-94 and PA11-95).

### Generation of a *Plcg2* knock-in mouse line that restores endogenous *Plcg2* expression in a Cre/loxP recombination-dependent manner

An EGFP-Pgk pA cassette of the *Plcg2*/EGFP knock-in targeting vector [4] was replaced with a cassette that contained a modified exon 2 of the *Plcg2* gene carrying a *loxP*-flanked dsRed-Express cDNA (Clontech/TaKaRa Bio, Shiga, Japan) with a BGH pA in the 5′ untranslated region. A *Pac*I-digested, linearized vector (20 μg) was introduced into 129 congenic *Plcg2*^+/al^ ES cells [4]. Mice were generated from two independent, correctly targeted clones, as assessed by PCR genotyping of the ESCs. Heterozygous *Plcg2*-floxed dsRed-Express knock-in mice without the neomycin-resistance gene were generated by crossing to the FLP-deleter strain FLP66 [34]. The mice were then backcrossed more than ten times to C57BL/6J mice before being used in this study.

### Immunohistochemistry and imaging

Specimens were fixed in Bouin’s fixative at 4°C for 2 days, dehydrated, and embedded in paraffin. Paraffin sections (5 μm) were prepared for HE staining and immunostaining. For immunohistochemistry, sections were antigen-retrieved in ImmunoSaver solution (Wako Pure Chemical Industries, Osaka, Japan) at 95°C for 45 min. All sections were incubated with 3% H_2_O_2_ in phosphate-buffered saline (PBS) or methanol prior to immunostaining. Sections were incubated with a primary antibody, followed by incubation with the Histofine reagent (Nichirei Biosciences, Tokyo, Japan). Before the detection of the primary/secondary antibody complexes against V-ATPase B1B2 shown in Fig 3C, sections were incubated with a biotinylated antibody against GFP for double immunostaining. A Streptavidin/Biotin blocking kit (Vector Laboratories, Burlingame, CA, USA) was used. Following the detection of the first antibody complexes, sections were incubated with 3% H_2_O_2_ to quench the peroxidase activity of the Histofine reagent and then incubated with streptavidin-HRP (NEN/PerkinElmer, Waltham, MA, USA). The TSA HRP detection system (NEN/PerkinElmer, Waltham, MA, USA) was used, and 4,6-diamidino-2-phenylindole (DAPI; Molecular Probes, Eugene, OR, USA) was used for nuclear staining. Fluorescence micrographs were acquired with a BioRevo BZ-9000 microscope (Keyence, Osaka, Japan). The following antibodies were used: rabbit polyclonal anti-PLCγ2 (SC-407), anti-PKCζ (SC-216), V-ATPase B1/B2 (SC-20943), and biotinylated rabbit polyclonal anti-GFP (SC-8334B; Santa Cruz Biotechnology, Santa Cruz, CA, USA); rabbit polyclonal anti-K5 (AF-138; Covance, Emeryville, CA, USA); rabbit polyclonal anti-Aqp9 (LS-C20770; LifeSpan Biosciences, Seattle, WA, USA); mouse monoclonal anti-ZO-1 (ZO1-1A12; Invitrogen, Carlsbad, CA, USA); and Cy3-conjugated mouse monoclonal anti-human αSMA (1A4; Sigma-Aldrich, St Louis, MO, USA).

For EGFP immunostaining on cryosections of mT/mG;*Vegfr3-Cre*39 mice, the mice were perfused with PBS containing 4% PFA. Tissues were dissected and immersion-fixed in 4% PFA in PBS at 4°C for 24 h, and then processed for cryo-sectioning. Frozen sections (10 μm) were rehydrated in PBS, and antigen retrieval was accomplished by incubation in PBS containing 0.1% trypsin and 0.5 mM EDTA at room temperature for 10 min. Before incubation with primary antibodies, all sections were incubated in PBS containing 3% H_2_O_2_ at room temperature for 15 min. Rat anti-GFP (Nacalai Tesque, Kyoto, Japan), Histofine reagent (Nichirei Biosciences, Tokyo, Japan), and the TSA/TSA PLUS HRP Detection System (NEN/PerkinElmer) were used.

Bright field and fluorescence micrographs were acquired in an Olympus SZX12 microscope equipped with a DP72 digital camera (Olympus, Tokyo, Japan) and a BioRevo BZ-9000 microscope (Keyence, Osaka, Japan). Micrographs in figures are representative of two independently stained sections or two independent specimens from three or more mice. Adjustment of the brightness and contrast levels using Adobe Photoshop was applied equally across entire images.

Lumens surrounded by the ZO-1-positive apical surface and epithelial cell areas between the ZO-1-positive apical surface and myoid cell layers in captured images were measured using a measurement module of BZ-9000 (Keyence, Osaka, Japan). Luminal space measurements were normalized by measurements of epithelial cell areas. Thirty ducts (ten ducts per epididymis, three epididymides) for each genotype were measured and analyzed. Statistical comparisons were made using the two-tailed paired Student’s t-test (α = 0.05).
